# Heavy Smoking Is More Strongly Associated with General Unhealthy Lifestyle than Obesity and Underweight

**DOI:** 10.1371/journal.pone.0148563

**Published:** 2016-02-24

**Authors:** Tina Lohse, Sabine Rohrmann, Matthias Bopp, David Faeh

**Affiliations:** Division of Chronic Disease Epidemiology, Epidemiology, Biostatistics and Prevention Institute (EBPI), University of Zurich, Zurich, Switzerland; Boston University, UNITED STATES

## Abstract

**Background:**

Smoking and obesity are major causes of non-communicable diseases. We investigated the associations of heavy smoking, obesity, and underweight with general lifestyle to infer which of these risk groups has the most unfavourable lifestyle.

**Methods:**

We used data from the population-based cross-sectional Swiss Health Survey (5 rounds 1992–2012), comprising 85,575 individuals aged≥18 years. Height, weight, smoking, diet, alcohol intake and physical activity were self-reported. Multinomial logistic regression was performed to analyse differences in lifestyle between the combinations of body mass index (BMI) category and smoking status.

**Results:**

Compared to normal-weight never smokers (reference), individuals who were normal-weight, obese, or underweight and smoked heavily at the same time had a poorer general lifestyle. The lifestyle of obese and underweight never smokers differed less from reference. Regardless of BMI category, in heavy smoking men and women the fruit and vegetable consumption was lower (e.g. obese heavy smoking men: relative risk ratio (RRR) 1.69 [95% confidence interval 1.30;2.21]) and high alcohol intake was more common (e.g. normal-weight heavy smoking women 5.51 [3.71;8.20]). In both sexes, physical inactivity was observed more often in heavy smokers and obese or underweight (e.g. underweight never smoking 1.29 [1.08;1.54] and heavy smoking women 2.02 [1.33;3.08]). A decrease of smoking prevalence was observed over time in normal-weight, but not in obese individuals.

**Conclusions:**

Unhealthy general lifestyle was associated with both heavy smoking and BMI extremes, but we observed a stronger association for heavy smoking. Future smoking prevention measures should pay attention to improvement of general lifestyle and co-occurrence with obesity and underweight.

## Introduction

Smoking and obesity are the most important modifiable risk factors of non-communicable diseases (NCD) [1–3]. Evidence is less clear for underweight [4,5]. However, similarly to obese individuals and smokers, it was shown that underweight individuals have an increased risk of premature death [6,7]. Investigations of the health impact of extreme body mass index (BMI) combined with smoking found that obese and underweight current smokers had the highest overall, cancer and cardiovascular disease mortality risk [8]. Non-smoking and maintaining healthy BMI, but also related risk factors such as healthy diet, low to moderate alcohol intake and physical activity offer substantial potential for the reduction of premature death and NCD burden in the population [9,10].

Unfavourable lifestyle factors are likely to occur coincidentally. Studies on how lifestyle factors are related and cluster revealed that smoking and educational level are driving factors, unfortunately they did not take BMI into account [11–13]. In affluent countries like Switzerland, obesity gained relevance as its prevalence was increased over the past decades, whereas the prevalence of smoking decreased in the general population [14,15]. However, it remains unknown whether this decrease occurred also in those most at risk (i.e. obese individuals) or mainly in healthier and health conscious people. Therefore we aimed to investigate the general lifestyle of obese individuals, heavy smokers, and obese heavy smokers to get a better understanding of the distribution of lifestyle risk factors. These populations are already at high risk of NCD and the coincidence with further unhealthy lifestyles would worsen their risk profile. We also included underweight in our analysis to contribute to the discussion on whether the increased mortality risk of underweight individuals is explained by associated lifestyle factors [6,16].

It was our first objective to compare the role of obesity, underweight and heavy smoking regarding the tendency of clustering with other NCD relevant lifestyle factors and with socio-demographic factors. Secondly, we aimed at investigating the temporal changes in the prevalence of the combination of obesity and underweight respectively with heavy smoking.

## Methods

### Population and data collection

The Swiss Health Survey (SHS) is a population-based cross-sectional survey conducted every 5 years since 1992 by the Swiss Federal Statistical Office [17]. Study samples were obtained by stratified random sampling out of a database containing all private household landline telephone numbers. This database was built with linkage of data from resident registries and telephone companies. Since 2012, an additional recruitment option was implemented. For those subjects who were randomly selected through resident registries and had no landline telephone number available, a letter was sent out to obtain contact information (landline or mobile telephone number) by prepaid answer postcard. Data was collected with telephone interview and self-administered questionnaire, additionally. The participation rate ranged from 71% in 1992 to 54% in 2012. For this study, we restricted the sample to individuals aged≥18 years.

The data collection and data storage for the SHS does not require formal approval by an ethical committee. This data collection is specifically permitted under Swiss law (Verordnung über die Durchführung von statistischen Erhebungen des Bundes vom 30. Juni 1993 (SR 431.012.1) and Verordnung über die eidgenössische Volkszählung vom 19. Dezember 2008 (SR 431.112.1)). Individuals invited to participate received a brief description of the study and could decline to participate or withdraw at any time. Participants’ responses were treated confidentially and aggregated anonymous responses were utilized for analyses presented herein.

### Outcome

Height, weight, and smoking status were self-reported by telephone interview (see S1 Table). BMI was calculated as weight in kilograms divided by the square of height in metres. We categorized BMI (kg/m^2^) into underweight <18.5, normal-weight ≥18.5–<25, overweight ≥25–<30, and obesity ≥30 [18]; smoking status into never, former, light (1–9 cigarettes per day), moderate (10–19), and heavy smokers (>19). Never smokers stated that they did not currently smoke and never regularly smoked during more than six months; former smokers reported not smoking currently but having smoked for more than 6 months during their life course. One cigarillo or pipe was counted as 2 cigarettes and 1 cigar as 4 cigarettes. The outcome variable had 20 categories, composed of the combination of BMI category and smoking status.

### Exposure and Covariates

We selected three lifestyle proxies in order to explore the general health behaviour. These were assessed by telephone interview and self-administered questionnaire: fruit and vegetable consumption, physical activity, and alcohol intake. In Switzerland, fruit and vegetable consumption—as healthy diet proxy—was associated with lower mortality, also in combination with other NCD factors [10]. For all 5 rounds of the SHS information on the number of days per week fruits and vegetables were consumed was available. We chose to categorize as closest to the "5-a-day" recommendation as possible [19]. Because of the inconsistency of the collected information across surveys, we had to choose a fairly crude categorisation. Fruit and vegetable consumption was combined in one binary variable that comprised the information on whether both fruits and vegetables were consumed daily or not. We previously showed the importance of leisure-time physical activity in avoiding premature death [20]; hence we included weekly leisure-time physical activity in the analysis. The variable was defined as the number of days per week a subject started to sweat during leisure time physical activity and was categorized as >2 days, 1–2 days, and none. Alcohol intake was categorized into low, moderate, and high based on its sex specific risk for adverse health consequences. For men, the cut-offs were <40 to <60g of alcohol per day, for women <20 to <40g. For 4,500 participants of the SHS 1992, information on alcohol intake was only available from the telephone interview. Because this information was not comparable to that obtained from the questionnaire, we added a missing category to the alcohol variable. Education was included as highest degree obtained and was categorized into mandatory (International Standard Classification of Education, ISCED 1–2), secondary II (ISCED 3–4), and tertiary (ISCED 5–8) [21].

### Statistical analysis

We pooled the data of the five SHS and included a survey variable in the model. All analyses were weighted to the general population of Switzerland [17] and stratified by sex. We stratified for sex because of existing evidence for variations between sexes which also were obvious in our data. Differences in prevalence of smoking status and BMI categories were substantial between men and women as well as in the distribution of the exposures (fruit and vegetables, physical activity, and alcohol intake). Furthermore, it is known that the reasons for smoking vary by sex and this may lead to differences in their association with further lifestyle factors [22,23]. We performed multinomial logistic regression (STATA command: mlogit) in order to examine whether heavy smoker, obese or underweight individuals as well as obese and underweight heavy smokers were prone to have additional unhealthy lifestyle factors, compared to normal-weight never smokers. Multinomial regression was used to investigate associations between a categorical outcome with more than 2 categories and the exposure. The outcome was defined as a categorical variable obtained by the combination of BMI category with smoking status category. All smoking-BMI-category-combinations (4x5) were included in the analyses. However, for this study, we focussed on the results for heavy smoking and BMI extremes (underweight and obesity) and their presentation. The three lifestyle variables were included in the model, as well as educational level, nationality, language region, survey (categorisation, see Tables 1 and 2), and age. We pooled the data of the 5 SHS rounds, which enabled us to investigate changes over time. This was done through interpreting the results of the survey variable that was entered into the multinomial regression model. The regression model provided relative risk ratios (RRR) [24].To assess the public health relevance, the absolute number of individuals per BMI category, smoking status, and selected combinations of smoking and BMI were estimated for Switzerland in 2012 by an extrapolation based on SHS 2012 and STATPOP 2012 (Statistics of population and households) [25]. All analyses were performed using STATA 13.1, College Station, TX, USA.

**Table 1:** Demographic characteristic, BMI category, smoking status, and survey.

	Men		Women	
Mean age	46.0*		47.9*	
	n	%*	n	%*
**Nationality**				
Swiss	28985	79.3	37450	83.4
Foreign	5256	20.7	5100	16.6
**Education**				
Tertiary	10814	31.0	6772	15.3
Secondary II	19412	56.0	26970	63.7
Mandatory	4015	12.0	8808	21.0
**Language region**				
German	22761	72.8	27532	71.2
French	9022	22.8	11686	24.0
Italian	2458	4.4	3332	4.8
**BMI category**				
Underweight	302	0.9	2628	6.3
Normal-weight	17748	52.4	27320	64.7
Overweight	13196	38.3	9263	21.5
Obese	2995	8.4	3339	7.5
**Smoking status**				
Never	14063	41.3	24431	58.4
Former	9515	26.9	8117	18.6
Light	2799	08.4	3236	7.8
Medium	3100	09.5	3524	8.1
Heavy	4764	13.9	3242	7.1
**Survey**				
1992	6003	18.8	7574	19.0
1997	5063	19.7	6518	19.8
2002	7389	18.8	9489	19.4
2007	7015	19.7	9064	19.5
2012	8771	23.0	9905	22.3
**N Total**	34241	100.0	42550	100.0

* weighted according to the general population of Switzerland.

**Table 2:** Estimated absolute numbers (N) and proportions (%*) for smoking status, BMI, and selected smoking-BMI-combinations in Switzerland 2012.

	Men		Women	
	n	%*	n	%*
**BMI**				
Underweight	25 771	0.8	193 971	5.8
Normal-weight	1 523 051	47.3	2 061 801	61.4
Overweight	1 296 723	40.3	780 803	23.2
Obese	372 537	11.6	322 836	9.6
**Smoking status**				
Never	1 466 277	45.6	1 999 815	59.5
Former	888 659	27.6	678 057	20.2
Light	263 114	8.2	271 525	8.1
Medium	295 869	9.2	262 438	7.8
Heavy	304 163	9.4	147 575	4.4
**Smoking—BMI—combination**				
Normal-weight/ Never smoker	756 523	23.5	1 213 258	36.1
Underweight/ Never smoker	15 485	0.5	119 422	3.6
Overweight/Never smoker	544 589	16.9	460 612	13.7
Obese/ Never smoker	148 484	4.6	202 028	6.0
Normal-weight/ Heavy smoker	138 192	4.3	85 537	2.5
Underweight/ Heavy smoker	3 861	0.1	10 566	0.3
Overweight/Heavy smoker	121 667	3.8	38 493	1.1
Obese/ Heavy smoker	41 910	1.3	14 299	0.4
**Total**	3 218 082	100.00	3 359 410	100.00

Extrapolation based on SHS 2012 (prevalence) and STATPOP 2012 (Statistics of population and households), permanent resident population aged ≥ 18 years, by sex.

* weighted according to the general population of Switzerland.

## Results

### Descriptive

Our analysis included 85,575 individuals. Table 1 shows the distribution of BMI and smoking status categories and demographic characteristics of the study participants by sex (BMI and smoking combinations see S2 Table, lifestyle exposures see S3 Table). Women were on average older than men, whereas there were only negligible differences in nationality and distribution over language regions. The proportion of individuals with tertiary education was twice as high in men compared to women. The prevalence of obesity was comparable in men and women, but the proportion of women with underweight was 6 times higher. Heavy smoking was twice as frequent in men compared to women.

**Fig 1:**
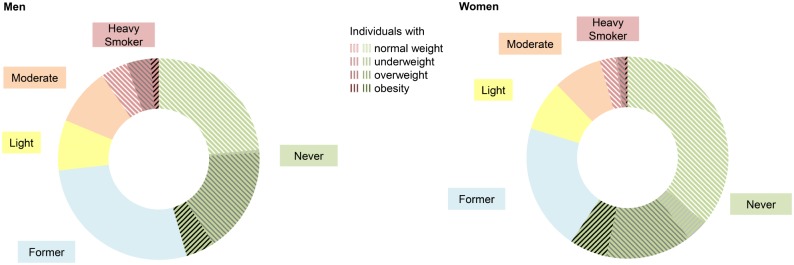
Prevalence of smokers by status additionally stratified by BMI for heavy and never smokers SHS 2012. Men n = 35,880 (missing n = 2,949) and women n = 44,142 (missing n = 2,604). BMI (Body Mass Index, kg/m^2^): underweight <18.5, normal-weight ≥18.5–<25, overweight ≥25–<30, obesity ≥30; Smoking status: never, former, light (1–9 cigarettes per day), moderate (10–19), heavy smoker (>19); SHS: Swiss Health Survey; Results are weighted according to the general population of Switzerland.

Fig 1 shows the distribution of smoking status by sex for Switzerland in 2012. For heavy and never smoking, combinations with BMI categories are presented in detail. Table 2 shows the corresponding proportions and estimated absolute numbers for Switzerland in 2012. In never and heavy smokers, the proportion of underweight and overweight individuals was comparable in both sexes. However, comparing male never with heavy smokers, the proportion of normal-weight individuals was smaller (never: 52 vs heavy: 45%) whereas the proportion of obese individuals was larger (never: 10 vs heavy: 14%). This difference was smaller in females (61 vs 57%; 10 vs 9%). Sex differences also existed with respect to the prevalence of the combination obesity plus heavy smoking. It was found to be 1.3% in men and 0.5% in women. Men were also more likely to be normal-weight heavy smokers. On the other hand, women were more often obese never smokers, underweight never and heavy smokers, respectively.

### Regression analysis

Compared to reference, i.e. normal-weight never smokers, individuals who were normal-weight, obese, or underweight and smoked heavily at the same time had a poorer lifestyle (Table 3); the lifestyle of obese and underweight never smokers differed less from reference. Heavy smokers (referred as smokers in this section) were observed to be more likely to have an unfavourable behaviour with respect to almost all modifiable lifestyle proxy factors, regardless of BMI. In contrast, physical inactivity was the only lifestyle factor that showed an association with being never smoker, except for hazardous alcohol intake in male obese never smokers. The association with the lifestyle factors was shown to be stronger in smokers compared to never smokers. Smokers of both sexes (except for heavy smoking obese women) were most likely to have a high alcohol intake.

**Table 3:** Lifestyle of obese and/or heavy smokers by sex, multinomial logistic regression: reference group normal-weight never smokers (adjusted for sociodemographic factors, age, and survey), weighted according to the general population of Switzerland.

	Normal-weight / Heavy smokers		Obese / Never smokers		Obese / Heavy smokers		Underweight / Never smokers		Underweight / Heavy smokers	
	RRR	(95% CI)	RRR	(95% CI)	RRR	(95% CI)	RRR	(95% CI)	RRR	(95% CI)
**Men**										
**Fruits and vegetables**										
Daily	1		1		1		1		1	
< Daily	**1.82**	**(1.62;2.05)**	1.18	(0.99;1.41)	**1.69**	**(1.30;2.21)**	1.21	(0.74;1.98)	1.85	(0.86;3.96)
**Physical activity, leisure**										
> 2	1		1		1		1		1	
1 to 2	**1.34**	**(1.16;1.56)**	1.23	(0.99;1.52)	1.34	(0.95;1.91)	0.91	(0.52;1.60)	1.11	(0.47;2.62)
Days per week										
None	**2.91**	**(2.50;3.37)**	**1.54**	**(1.24;1.89)**	**3.31**	**(2.38;4.62)**	1.29	(0.72;2.30)	**3.35**	**(1.43;7.87)**
Alcohol*										
Low	1		1		1		1		1	
Moderate	**3.35**	**(2.59;4.35)**	1.18	(0.75;1.86)	**2.15**	**(1.29;3.60)**	1.06	(0.20;5.55)	**5.33**	**(1.59;17.87)**
High	**4.75**	**(3.58;6.31)**	**2.00**	**(1.23;3.25)**	**4.29**	**(2.60;7.07)**	0.24	(0.03;1.76)	**6.83**	**(2.37;19.68)**
Missing	**1.55**	**(1.20;2.00)**	1.36	(0.83;2.22)	1.16	(0.65;2.10)	1.83	(0.53;6.27)	0.62	(0.17;2.18)
**Education**										
Tertiary	1		1		1		1		1	
Secondary II	**2.04**	**(1.79;2.33)**	**1.74**	**(1.43;2.11)**	**2.86**	**(2.06;3.96)**	**1.93**	**(1.17;3.20)**	**6.97**	**(2.87;16.94)**
Mandatory	**2.19**	**(1.79;2.69)**	**2.67**	**(2.02;3.52)**	**3.26**	**(2.13;4.99)**	**3.53**	**(1.77;7.05)**	**11.67**	**(3.94;34.61)**
**Nationality**										
Swiss	1		1		1		1		1	
Foreign	**1.43**	**(1.23;1.67)**	**1.60**	**(1.25;2.05)**	**1.51**	**(1.08;2.13)**	1.19	(0.66;2.12)	1.11	(0.44;2.82)
Language region**										
German	1		1		1		1		1	
French	1.01	(0.89;1.15)	0.88	(0.73;1.07)	**0.73**	**(0.55;0.99)**	1.31	(0.77;2.23)	1.24	(0.62;2.48)
Italian	0.83	(0.66;1.04)	1.07	(0.80;1.44)	1.00	(0.64;1.55)	1.41	(0.67;2.95)	0.81	(0.27;2.45)
**Survey**										
1992	1		1		1		1		1	
1997	1.03	(0.86;1.23)	1.19	(0.84;1.70)	1.10	(0.72;1.69)	1.83	(0.79;4.21)	0.58	(0.22;1.52)
2002	0.86	(0.72;1.03)	**1.58**	**(1.13;2.22)**	1.19	(0.79;1.79)	2.31	(0.99;5.39)	0.81	(0.35;1.90)
2007	**0.60**	**(0.49;0.73)**	**2.12**	**(1.53;2.94)**	0.95	(0.61;1.48)	1.26	(0.51;3.08)	0.40	(0.13;1.25)
2012	**0.47**	**(0.39;0.58)**	**2.54**	**(1.85;3.48)**	1.15	(0.76;1.73)	1.38	(0.58;3.28)	0.52	(0.18;1.50)
**Age**	1.00	(1.00;1.00)	**1.04**	**(1.03;1.04)**	**1.02**	**(1.01;1.02)**	**0.97**	**(0.95;0.99)**	**0.97**	**(0.95;1.00)**
**n**		2538		1037		434		121		66
**Women**										
**Fruits and vegetables**										
Daily	1		1		1		1		1	
< Daily	**2.48**	**(2.19;2.80)**	0.96	(0.84;1.10)	**1.71**	**(1.22;2.40)**	1.14	(0.98;1.33)	**1.91**	**(1.42;2.56)**
**Physical activity, leisure**										
> 2	1		1				1		1	
1 to 2	0.86	(0.73;1.02)	1.01	(0.85;1.21)	0.90	(0.54;1.51)	0.96	(0.80;1.15)	0.92	(0.59;1.44)
Days per week										
None	**1.75**	**(1.49;2.06)**	**1.55**	**(1.32;1.83)**	**2.18**	**(1.38;3.43)**	**1.29**	**(1.08;1.54)**	**2.02**	**(1.33;3.08)**
Alcohol*										
Low	1		1		1		1		1	
Moderate	**3.50**	**(2.76;4.43)**	0.81	(0.53;1.24)	0.55	(0.22;1.39)	1.07	(0.70;1.63)	**3.42**	**(2.06;5.69)**
High	**5.51**	**(3.71;8.20)**	0.83	(0.40;1.72)	2.19	(0.64;7.49)	0.91	(0.39;2.08)	**5.90**	**(2.54;13.68)**
Missing	**1.47**	**(1.14;1.90)**	1.05	(0.76;1.46)	0.59	(0.18;1.94)	0.95	(0.69;1.32)	1.72	(0.92;3.22)
**Education**										
Tertiary	1		1				1		1	
Secondary II	**1.91**	**(1.59;2.28)**	**1.78**	**(1.44;2.19)**	**1.83**	**(1.04;3.24)**	**0.78**	**(0.66;0.93)**	1.51	(0.96;2.37)
Mandatory	**2.07**	**(1.67;2.57)**	**3.43**	**(2.73;4.30)**	**4.28**	**(2.33;7.84)**	**0.69**	**(0.54;0.87)**	1.62	(0.95;2.79)
**Nationality**										
Swiss	1		1		1		1		1	
Foreign	**0.75**	**(0.62;0.90)**	**1.37**	**(1.15;1.65)**	0.67	(0.40;1.13)	**0.69**	**(0.55;0.87)**	0.63	(0.39;1.00)
Language region**										
German	1		1		1		1		1	
French	**1.30**	**(1.14;1.47)**	**0.84**	**(0.73;0.96)**	0.86	(0.60;1.23)	**1.28**	**(1.10;1.50)**	**1.76**	**(1.30;2.39)**
Italian	0.97	(0.79;1.19)	**0.68**	**(0.55;0.85)**	0.79	(0.43;1.43)	**1.58**	**(1.26;1.99)**	0.94	(0.58;1.54)
**Survey**										
1992	1		1		1		1		1	
1997	**1.23**	**(1.03;1.47)**	**1.37**	**(1.09;1.74)**	**2.56**	**(1.32;4.97)**	0.81	(0.65;1.02)	1.28	(0.81;2.01)
2002	1.01	(0.84;1.21)	**1.58**	**(1.26;1.99)**	**2.31**	**(1.22;4.36)**	0.88	(0.71;1.10)	1.03	(0.66;1.63)
2007	0.93	(0.76;1.13)	**1.84**	**(1.46;2.31)**	1.90	(0.96;3.75)	**0.77**	**(0.61;0.96)**	0.93	(0.56;1.54)
2012	**0.56**	**(0.46;0.70)**	**2.07**	**(1.66;2.59)**	**2.09**	**(1.05;4.15)**	0.93	(0.75;1.15)	**0.50**	**(0.30;0.82)**
**Age**	**0.98**	**(0.97;0.98)**	**1.03**	**(1.02;1.03)**	0.99	(0.98;1.00)	**0.98**	**(0.97;0.98)**	**0.97**	**(0.96;0.98)**
**n**		2085		1980		227		1347		313

*Cut-offs: men <40g and <60g and women <20g and <40g of alcohol per day;

**German included Romansh; results shown only for selected combinations of BMI and smoking status.

Missing: BMI n = 1258, smoking status n = 4382, fruits and vegetables n = 1571, physical activity n = 3555, alcohol n = 4966.

The results for smokers by the investigated BMI categories are described in depth as follows. Male normal-weight and obese smokers were likely to have an infrequent fruit and vegetable consumption, low physical inactivity level, and high alcohol intake. For example in men, if an individual reported a low fruit and vegetable consumption, the relative risk ratio for being an obese smoker relative to normal-weight never smoker would be expected to be increased (RRR 1.69 [1.30;2.21]) compared to an individual having a high fruit and vegetable consumption. In underweight smoking men, significant associations were found for physical inactivity and high risk alcohol intake, despite the small stratum size and therefore wide confidence intervals. Female normal-weight and underweight smokers were likely to have an unfavourable behaviour in all three lifestyle factors. Obese smoking women were more likely to have infrequent fruit and vegetable consumption and high alcohol intake. In contrast to men, women who smoked and/or were underweight or obese were more likely to be physically inactive.

Only a selection of the smoking/BMI combination groups is shown. To briefly summarize the results for the remaining 14 outcome categories, we found that individuals in these categories were less likely to have low fruit and vegetable consumption, high alcohol intake, and physical inactivity, compared to those individuals in the categories with heavy smoking and extreme BMI (not shown). Only overweight heavy smokers showed similar poor behaviour in the three lifestyle variables investigated. In addition, we observed that the lifestyle tended to deteriorate, the more an individual smoked.

Socio-demographic adjustment variables were strongly associated with the combination of heavy smoking and obesity or underweight. In general, individuals with lower educational level were more likely to have an extreme BMI and being a smoker. However, this association was reversed in underweight women. Male foreign nationals were more likely to be normal-weight or obese smokers and obese never smokers, respectively. Being female Swiss national was associated with being normal-weight smoker or underweight never smoker, whereas foreign nationals were more likely to be obese never smokers. A significant impact of language region was observed mainly in women. Compared to women living in the German speaking part, women from the French and Italian speaking region were more likely to be underweight and less likely to be obese. Moreover, those from the French speaking part were more likely to be underweight smokers.

A decrease in the prevalence of heavy smokers between 1992 and 2012 was observed in those with normal-weight, especially in men (Table 3). Furthermore, the results suggest that the prevalence of the combination of heavy smoking with underweight and obesity changed in women only; it increased in female obese heavy smokers and decreased female underweight heavy smokers. The prevalence of obese never smokers increased in both sexes.

## Discussion

### Main results

In this study, we investigated how heavy smoking and extreme BMI as well as the combination of both were associated with other NCD risk factors. Heavy smokers were more likely to have a poor diet, high alcohol intake and low level of physical activity than obese or underweight individuals. While the prevalence of smoking decreased over time in combination with normal-weight, it increased in combination with obesity in women.

### Clustering of lifestyle factors

In line with our results, studies on clustering effects of unfavourable lifestyle factors in adults emphasise the role of smoking as driving factor [11,26]. The association of smoking was observed to be particularly strong with high alcohol intake [27]. Clustering effects of unhealthy lifestyles were reported to be more likely in individuals with a low educational level [12,13]. In our study, this was consistently shown in men, i.e. smoking and high/low BMI were associated with low educational level. In contrast, this association was reversed in women, i.e. underweight was associated with high educational level. Others showed that men and especially women with higher socioeconomic status were more concerned about their body weight and made more efforts to control it [28,29]. Interestingly, unhealthy behaviours were found to cluster stronger than healthy behaviours [30]. We are not aware of other studies performing a comparative analysis of the lifestyle of heavy smokers, obese, and underweight individuals. However, a study looking at age-specific lifestyle risk factors for obesity observed that young and middle-aged obese adults were frequently physically inactive; in older obese adults, poor eating habits were identified as an additional risk factor [31]. So far, much less has been reported about the lifestyle of underweight individuals because in developed countries the prevalence and, therewith, public health relevance is lower compared to obesity [32]. Reverse causation due to smoking and pre-existing disease has to be taken into account when studying health effects of underweight, e.g. mortality [33,34]. Our findings suggest that underweight never smoking women were at risk for physical inactivity, which could be either an attitude or indicating an underlying disease.

### Prevalence trends

We found that the prevalence of normal-weight heavy smokers decreased in Switzerland between 1992 and 2012 in both sexes. Across Europe, considerable differences in smoking prevalence exist; eastern and low income countries as well as countries with less advanced tobacco control policies have highest smoking prevalence [35]. An estimation of smoking prevalence worldwide showed that it is especially high among men in South, Southeast, and East Asia, e.g. more than 50% in Russia and Indonesia [14]. Our results show that in Switzerland the decrease in smoking prevalence mainly occurred in those with normal BMI; amongst obese individuals, the smoking prevalence stagnated (men) or even increased (women). This suggests that clustering of unhealthy lifestyles persisted or accentuated over time in part of the population, low socioeconomic status was shown to be an important factor explaining this effect [36–38].

### Public health relevance

Our results support the notion of smoking as a key determinant of an unhealthy lifestyle. In light of the ongoing clustering of smoking with other unfavourable lifestyle factors, efforts aimed at reducing tobacco use in the population need to be intensified. This is supported by the recent trends of cancer death in women; in Europe lung cancer is the leading cause, thus superseded breast cancer [39]. In Switzerland, previous efforts led to a decrease of the smoking prevalence in general, but a decrease was only observed in the normal-weight part of the population. Because obese and underweight smokers have a particularly problematic health risk pattern, a decrease in smoking prevalence would be even more important than in normal-weight individuals. In Switzerland, obesity contributes to an excess in death of about 7% and costs of 8 billion Swiss Francs; smoking of about 12% and 10 billion Swiss Francs [40–43]. As shown by Li et al. [44], non-smoking and maintaining healthy body weight are the lifestyles with the greatest potential to reduce the number of premature deaths and should therefore be the main target of public health strategies improving lifestyle.

### Strengths and Limitations

The SHS is a comprehensive survey collecting data on major lifestyle risk factors through a large representative sample of the general population. The large sample size allowed for the analysis of lifestyle factors in defined risk groups based on BMI category and smoking status. Potential confounders were included in the analysis, especially education. The repeated assessment (the SHS is conducted every 5 years) provided insights into changes over time in prevalence of the considered risk groups.

The participation rate decreased over the 5 SHS from 71% in 1992 to 53% in 2012 [17,45]. Two measures were implemented to account for this decrease in participation rate. First, in 2012 efforts were intensified to include persons having no landline telephone number available in the database, by sending out prepaid answer postcards to obtain further contact information to conduct the telephone interview. Second, analysis of the SHS data has to be done by applying weighting according to the general population of Switzerland. Nevertheless, it is likely that participants tend to be healthier than non-participants [46]. Self-reporting on lifestyle variables made non-differential misclassification of those variables more likely. Only a short questionnaire was used to evaluate fruit and vegetable consumption, alcohol intake and physical activity [47,48]. To draw conclusions, for example on the adherence to the “5-a-day” recommendation, more detailed information would be needed. The assessment of fruit and vegetable consumption was even aggravated by the fact that the collected information changed over the course of surveys and, therefore, it was necessary to use a dichotomized variable. BMI was shown to be underestimated in obese and overestimated in underweight [49]. For underweight, effect estimates were imprecise as strata size was small. Finally, due to the cross-sectional study design no causal relationships can be inferred.

## Conclusion

Both heavy smoking and BMI extremes were associated with unhealthy general lifestyle, rendering them particularly vulnerable for NCDs. However, the relationship was stronger for heavy smoking than for obesity and underweight. Smoking prevention measures should pay special attention to improvement of general lifestyle and co-occurrence with obesity and underweight. Future research in this area should focus on how lifestyle factors are interacting in the development of NCDs, i.e. looking at lifestyle patterns rather than single lifestyle risk factors. In addition, investigating the role of lifestyle factors using a life course approach may help to deepen the understanding of their association with NCDs.

## Supporting Information

S1 Table Data Collection on Height, Weight, and Smoking Status, Swiss Health Survey 1992 to 2012. (DOCX)

S2 Table Distribution of selected BMI category and Smoking Status Combinations, SHS 1992 to 2012, *weighted according to the Swiss general population. (DOCX)

S3 Table Distribution of fruit and vegetable consumption, physical activity, and alcohol intake, SHS 1992 to 2012, *weighted according to the Swiss general population. (DOCX)
