# Supplementary material for: Heavy Smoking Is More Strongly Associated with General Unhealthy Lifestyle than Obesity and Underweight
Source: PLoS One. 2016 Feb 24;11(2):e0148563. doi: 10.1371/journal.pone.0148563 (PMC4765891; doi:10.1371/journal.pone.0148563)
Supplement: S1 Table — (DOCX) [file pone.0148563.s001.docx]

**S1 Table.** **Data Collection on Height, Weight, and Smoking Status, Swiss Health Survey 1992 to 2012.**

| **Height and weight** | | | | | | | | | |
| --- | --- | --- | --- | --- | --- | --- | --- | --- | --- |
| **Nr.** | **Fragen** | **Thema** | **Modul** | **1992** | **1997** | **2002** | **2007** | **2012** | **Quelle / Bemerkungen** |
| 23.00 | Wieder an alle  Können Sie mir sagen, wie gross Sie ohne Schuhe sind? ---------------------------------------------  - Zentimeter *(3-stellig)* _ _ _  ---------------------------------------------  - Weiss nicht (-1)  - Keine Antwort (-2) | Gesundheitszustand Körpermasse TGEZU01b | Kern | 1 | 1 | 1 | 1 | 1 | IGIP tel. 54.00  *SGB07: TGEZU01a entfernt, da überflüssig.* |
| 23.10 | Und wie schwer sind Sie ohne Kleider?  Bei schwangeren Frauen (22.00 = 1):  Wie schwer sind Sie am Anfang von der Schwangerschaft gewesen?  ---------------------------------------------  - Kilos *(3-stellig)* _ _ _  ---------------------------------------------  - Weiss nicht (-1)  - Keine Antwort (-2) | Gesundheitszustand Körpermasse TGEZU02b | Kern | 1 | 1 | 1 | 1 | 1 | IGIP tel. 55.00  *SGB07: TGEZU02a entfernt, da überflüssig* |
| **Smoking** | | | | | | | | | |
| 39.00 | Rauchen Sie, wenn auch nur selten?  ----------------------------------------------  - Ja 1  - Nein 2 weiter zu 39.20  ----------------------------------------------  - Keine Antwort.................... (-2) weiter zu 39.20 | Tabakkonsum TTAKO01 | Kern | 1 | 1 | 1 | 1 | 1 | Anal. IGIP tel. 28.00 |
| 39.01 | Rauchen Sie täglich?  ----------------------------------------------  - Ja 1  - Nein 2  ----------------------------------------------  - Keine Antwort.................... (-2) | Tabakkonsum  TTAKO21 | Kern | 0 | 0 | 0 | 1 | 1 | EHIS angepasst |
| 39.10 | Was rauchen Sie?  *INT: Vorlesen!*  ---------------------------------------------  - Zigaretten Ja=1 Nein=2 Keine Antwort=(-2)  - Zigarren Ja=1 Nein=2 Keine Antwort=(-2)  - Cigarillos Ja=1 Nein=2 Keine Antwort=(-2)  - Pfeife Ja=1 Nein=2 Keine Antwort=(-2)  - Wasserpfeife Ja=1 Nein=2 Keine Antwort=(-2) | Tabakkonsum  TTAKO02a  TTAKO02b  TTAKO02c  TTAKO02d  TTAKO02e | Kern | 1 | 1 | 1 | 1 | 1 | IGIP tel. 28.10  *SGB07 mit TTAKO02e ergänzt* |

**S1 Table, continued. Data Collection on Height, Weight, and Smoking Status; Swiss Health Survey 1992 to 2012.**

| 39.11 | Personen, die Zigaretten rauchen (ttako02a/39.10=1)  Wieviele Zigaretten rauchen Sie im Durchschnitt pro Tag?  *INT: 1 Paket = 20 Zigaretten / 1/2Paket =10 Zigaretten*  ---------------------------------------------  - Zigaretten *(2-stellig)* _ _  - Weniger als 1 pro Tag 00  ---------------------------------------------  - Keine Antwort (-2) | Tabakkonsum  TTAKO03 | Kern | 1 | 1 | 1 | 1 | 1 | IGIP tel. 28.11 |
| --- | --- | --- | --- | --- | --- | --- | --- | --- | --- |
| 39.12 | Personen, die Zigarren rauchen (ttako02b/39.10=1)  Wie viele Zigarren rauchen Sie im Durchschnitt pro Tag?  ---------------------------------------------  - Zigarren *(2-stellig)* _ _  - Weniger als 1 pro Tag 00  ---------------------------------------------  - Keine Antwort (-2) | Tabakkonsum  TTAKO04 | Kern | 1 | 1 | 1 | 1 | 1 | IGIP tel. 28.12 |
| **Nr.** | **Fragen** | **Thema** | **Modul** | **1992** | **1997** | **2002** | **2007** | **2012** | **Quelle / Bemerkungen** |
| 39.13 | Personen, die Cigarillos rauchen (ttako02c/39.10=1)  Wie viele Cigarillos rauchen Sie im Durchschnitt pro Tag? ---------------------------------------------  - Cigarillos *(2-stellig)* _ _  - Weniger als 1 pro Tag 00  ---------------------------------------------  - Keine Antwort (-2) | Tabakkonsum  TTAKO05 | Kern | 1 | 1 | 1 | 1 | 1 | IGIP tel. 28.13 |
| 39.14 | Personen, die Pfeife rauchen (ttako02d/39.10=1)  Wie viele Pfeifen rauchen Sie im Durchschnitt pro Tag? ---------------------------------------------  - Pfeifen *(2-stellig)* _ _  - Weniger als 1 pro Tag 00  ---------------------------------------------  - Keine Antwort (-2) | Tabakkonsum  TTAKO06 | Kern | 1 | 1 | 1 | 1 | 1 | IGIP tel. 28.14 |
| **Nr.** | **Fragen** | **Thema** | **Modul** | **1992** | **1997** | **2002** | **2007** | **2012** | **Quelle / Bemerkungen** |
| 39.20 | Nur Nichtraucher/innen (+KA) (TTAKO01/39.00=2, -2).  Raucher/innen weiter zu Frage 39.30  Haben Sie je regelmässig während mehr als 6 Monaten geraucht?  ---------------------------------------------  - Ja 1  - Nein 2 weiter zu 39.50  ---------------------------------------------  - Keine Antwort (-2) weiter zu 39.50 | Tabakkonsum  TTAKO07 | Kern | 1 | 1 | 1 | 1 | 1 | IGIP tel. 28.30 |
