# Supplementary material for: Heavy Smoking Is More Strongly Associated with General Unhealthy Lifestyle than Obesity and Underweight
Source: PLoS One. 2016 Feb 24;11(2):e0148563. doi: 10.1371/journal.pone.0148563 (PMC4765891; doi:10.1371/journal.pone.0148563)
Supplement: S2 Table — (DOCX) [file pone.0148563.s002.docx]

**S2 Table.** **Distribution of selected BMI category and Smoking Status Combinations, SHS 1992 to 2012, *weighted according to the Swiss general population.**

|  |  | **Men** | |  | **Women** | |
| --- | --- | --- | --- | --- | --- | --- |
|  |  | **n** | **%*** |  | **n** | **%*** |
| **Smoking - BMI - combination** |  |  |  |  |  |  |
| Normal-weight/ Never smoker |  | 9106 | 25.4 |  | 16850 | 38.5 |
| Normal-weight/ Heavy smoker |  | 2595 | 6.8 |  | 2131 | 4.2 |
| Obese/ Never smoker |  | 1124 | 3.0 |  | 2108 | 4.6 |
| Obese/ Heavy smoker |  | 441 | 1.2 |  | 234 | 0.4 |
| Underweight/ Never smoker |  | 273 | 0.9 |  | 1651 | 4.0 |
| Underweight/ Heavy smoker |  | 69 | 0.2 |  | 328 | 0.6 |
| **Total** |  | 37152 | 100.00 |  | 45395 | 100.00 |
