# Supplementary material for: Heavy Smoking Is More Strongly Associated with General Unhealthy Lifestyle than Obesity and Underweight
Source: PLoS One. 2016 Feb 24;11(2):e0148563. doi: 10.1371/journal.pone.0148563 (PMC4765891; doi:10.1371/journal.pone.0148563)
Supplement: S3 Table — (DOCX) [file pone.0148563.s003.docx]

**S3 Table. Distribution of fruit and vegetable consumption, physical activity, and alcohol intake, SHS 1992 to 2012, *weighted according to the Swiss general population.**

|  |  | **Men** | |  | **Women** | |
| --- | --- | --- | --- | --- | --- | --- |
|  |  | **n** | **%*** |  | **n** | **%*** |
| **Fruit and vegetable consumption** |  |  |  |  |  |  |
| Daily |  | 19022 | 50.9 |  | 32409 | 70.5 |
| < Daily |  | 18875 | 49.1 |  | 13698 | 29.5 |
| **Physical activity, leisure time, days per week** |  |  |  |  |  |  |
| >2 |  | 11068 | 30.8 |  | 10449 | 23.7 |
| 1 to 2 |  | 13501 | 37.2 |  | 15605 | 35.0 |
| None |  | 12175 | 32.0 |  | 19222 | 41.3 |
| **Alcohol intake** |  |  |  |  |  |  |
| Low |  | 33465 | 86.0 |  | 42180 | 90.1 |
| Moderate |  | 1622 | 4.0 |  | 1531 | 3.1 |
| High |  | 1343 | 3.2 |  | 468 | 1.0 |
| Missing |  | 2399 | 6.8 |  | 2567 | 5.8 |
| **Total** |  | 37152 | 100.00 |  | 45395 | 100.00 |
